# Osteoid osteoma: which is the best mininvasive treatment option?

**DOI:** 10.1007/s00590-021-02946-w

**Published:** 2021-04-11

**Authors:** Anna Parmeggiani, Claudia Martella, Luca Ceccarelli, Marco Miceli, Paolo Spinnato, Giancarlo Facchini

**Affiliations:** 1 Diagnostic and Interventional Radiology, IRCCS Istituto Ortopedico Rizzoli, Via G.C.Pupilli, 1-40136 Bologna, Italy; 2 U.O. Di Radiologia, Ospedale degli Infermi, Azienda AUSL Della Romagna, Faenza, Italy

**Keywords:** Osteoma, Osteoid, Ablation techniques, Magnetic resonance imaging, Interventional, Radiography

## Abstract

Osteoid osteoma is the third most common benign bone tumor, with well-known clinical presentation and radiological features. Although surgical excision has been the only therapeutic option for a long time, to date it has been replaced by minimally invasive techniques, which proved satisfactory success rates and low complication occurrence. Therefore, the purpose of this literature review was to describe the main updates of these recent procedures in the field of interventional radiology, with particular attention paid to the results of the leading studies relating to the efficacy, complications, and recurrence rate. Nevertheless, this study aimed to analyze the peculiarities of each reported technique, with specific focus on the possible improvements and pitfalls. Results proved that all mininvasive procedures boast a high success rate with slight number of complications and a low recurrence rate. Radiofrequency ablation is still considered the gold standard procedure for percutaneous treatment of osteoid osteoma, and it has the possibility to combine treatment with a biopsy. Interstitial laser ablation’s advantages are the simplicity of use and a lower cost of the electrodes, while cryoablation allows real-time visualization of the ablated zone, increasing the treatment safety. Magnetic resonance-guided focused ultrasound surgery is the most innovative non-invasive procedure, with the unquestionable advantage to be radiation free.

## Introduction

Osteoid osteoma (OO) is the third most common benign bone tumor, representing approximately 3% of all primary bone tumors, and it mainly occurs between 5 and 25 years with a prevalence in the male population. The most affected site is the lower limb, particularly femur and tibia, and about 10% of osteoid osteomas arise in the vertebrae, primarily in the posterior elements [1–3].

Osteoid osteoma is radiologically and histologically characterized by a central hypervascular nidus of osteoblastic cells with a maximum diameter of 2 cm, surrounded by a sclerotic reaction, with low growth tendency [3].

Osteoid osteoma clinical presentation is extremely distinctive, represented by pain of variable intensity at the site of onset, that typically worsens during the night and improves with the administration of non-steroidal anti-inflammatory drugs [4].

Typical radiographic findings of osteoid osteoma include an intracortical radiolucent nidus associated with cortical thickening and reactive sclerosis, that at computed tomography (CT) examination manifests as a well-defined area of low attenuation with possibly central high attenuation focal spot representing mineralized osteoid [5].

Osteoid osteoma magnetic resonance imaging (MRI) findings include a hyperintense nidus with extensive adjacent bone marrow edema on fat-saturated sequences, characterized by contrast-enhancement after contrast medium administration [6]. Additionally, MRI better assesses OO relationship with nearby vascular and nerve structures, and it is a useful tool in the follow-up after percutaneous treatment [6, 7]. In fact, although CT can demonstrate the progressive dimensional nidus reduction and the cortical thickening resulting from ablation procedure, it is unable to evaluate post-procedural bone marrow edema and modifications in the adjacent soft tissues [8].

Technetium-99-labeled bone scintigraphy provides a metabolic assessment of the lesion, reflecting the radiotracer concentration at the site of increased osteoblastic activity, thus possibly supporting CT in the precise osteoid osteoma’s localization before treatment [9].

Although OO is a benign and self-limiting lesion, its spontaneous regression can take many years, causing patient’s protracted pain and forcing continuous and harmful use of anti-inflammatory drugs, with a significant deterioration in quality of life [2].

Surgical excision has been the only therapeutic option for a long time, albeit often associated with interventions, prolonged hospitalization, and significant risk of local recurrence in case of suboptimal resection. Over the years, less invasive treatment techniques have been developed with satisfactory success rates and improved post-treatment period [4].

This literature review aims to describe the main updates of these recent procedures in the field of interventional radiology, with particular attention paid to the results of the leading studies relating to the efficacy, complications, and recurrence rate, analyzing the peculiarities of each reported technique, with specific focus on the possible improvements and pitfalls and, related to the literature, considering other parameters like imaging guidance, dosimetry, and costs.

The main characteristics of each of the percutaneous techniques described are summarized in Table 1.

**Table 1:** Main differences of each mininvasive technique analyzed

Treatment	Biopsy	Ionizing radiation	Ablated zone identification	Locations full accessibility
Radiofrequency ablation (RFA)	+	+	−	+
Interstitial laser ablation (ILA)	+	+	−	+
Microwave ablation (MWA)	+	+	−	+
Cryoablation	+	+	+	+
Magnetic resonance-guided focused ultrasound (MRg-FUS)	−	−	−	−

## Radiofrequency ablation (RFA)

Rosenthal was the first to successfully pave the way toward the use of radiofrequency for the treatment of OO. Since then, this technique has achieved excellent results in terms of efficacy and safety, so that percutaneous CT-guided radiofrequency ablation (CT-RFA) has become the gold standard therapeutic technique, replacing surgical treatment [10–12].

Furthermore, in comparison to surgery, RFA allows to reach difficult anatomical sites and provides shorter operation and hospitalization periods, with consequent costs reduction [12]. Specifically, Göksel et al. [13] carried out a comparative study on 24 patients with OO, 11 of which (45.83%) underwent surgical curettage, while 13 (54.17%) received RFA. Mean operation length of the surgery group was 69.5 min, with mean hospital stay of 1.3 days, while they were significantly shorter in the RFA group with mean operation length of 49.6 min and mean hospital stay of 0.3 days.

In technical terms, RFA is performed under CT guidance, necessary for lesion localization and electrode placement evaluation. Patients are usually under general, spinal, or propofol-induced anesthesia; local anesthesia alone is often associated with inadequate pain control [14].

**Fig. 1:**
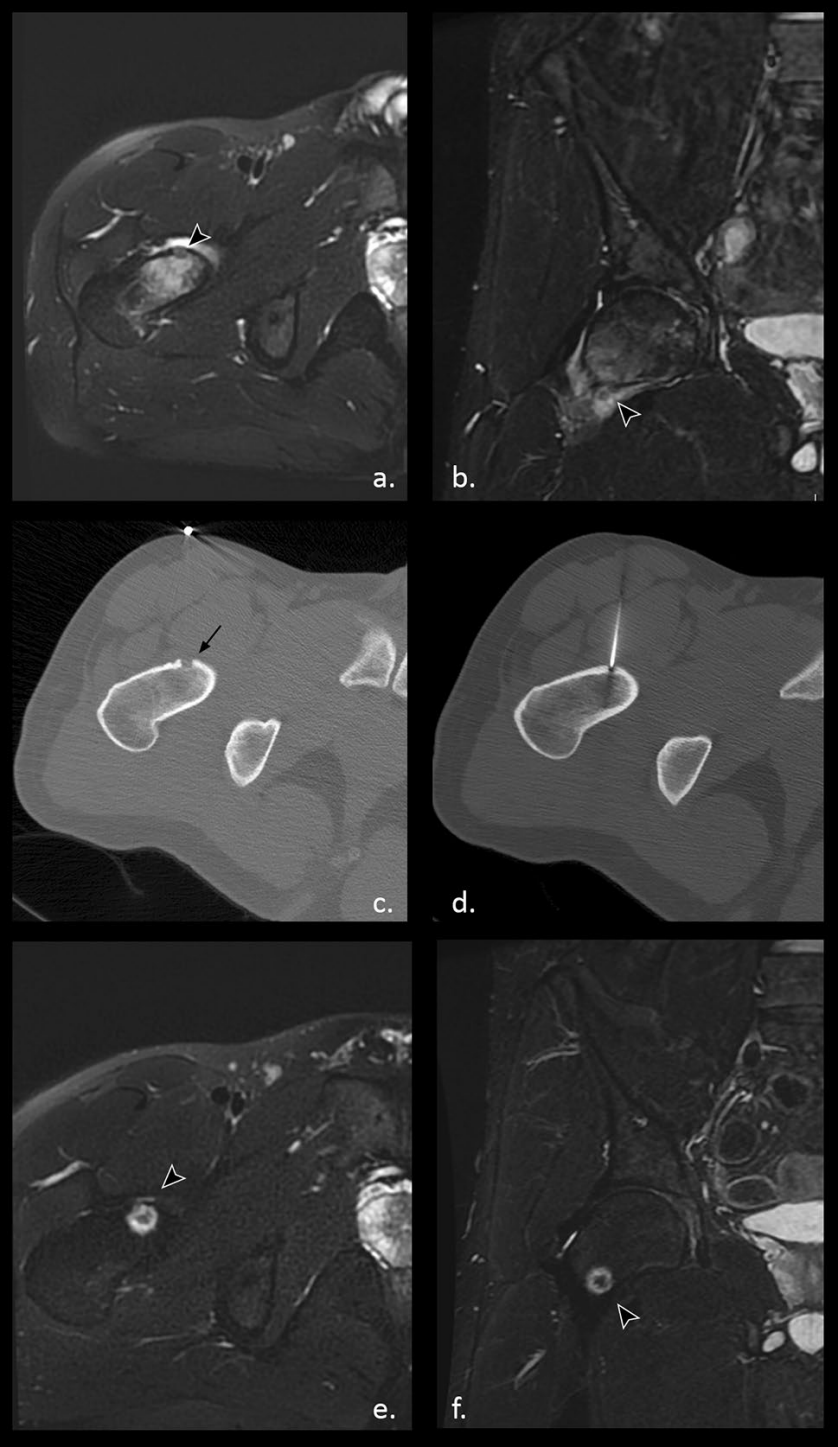
Osteoid osteoma of the right femoral neck. Axial (**a**) and coronal (**b**) MRI sequences weighted in STIR reveal the central nidus of the lesion (arrowhead) surrounded by extensive spongy bone edema and moderate joint effusion. **c** Axial CT scan shows the intracortical radiolucent nidus (black arrow) and the above cutaneous metal trace; **d** needle electrode positioning during radiofrequency thermal ablation procedure. Axial (**e**) and coronal (**f**) follow-up MRI sequences weighted in STIR after RFA demonstrate a sharp rim of post-procedural edema around the nidus

**Fig. 2:**
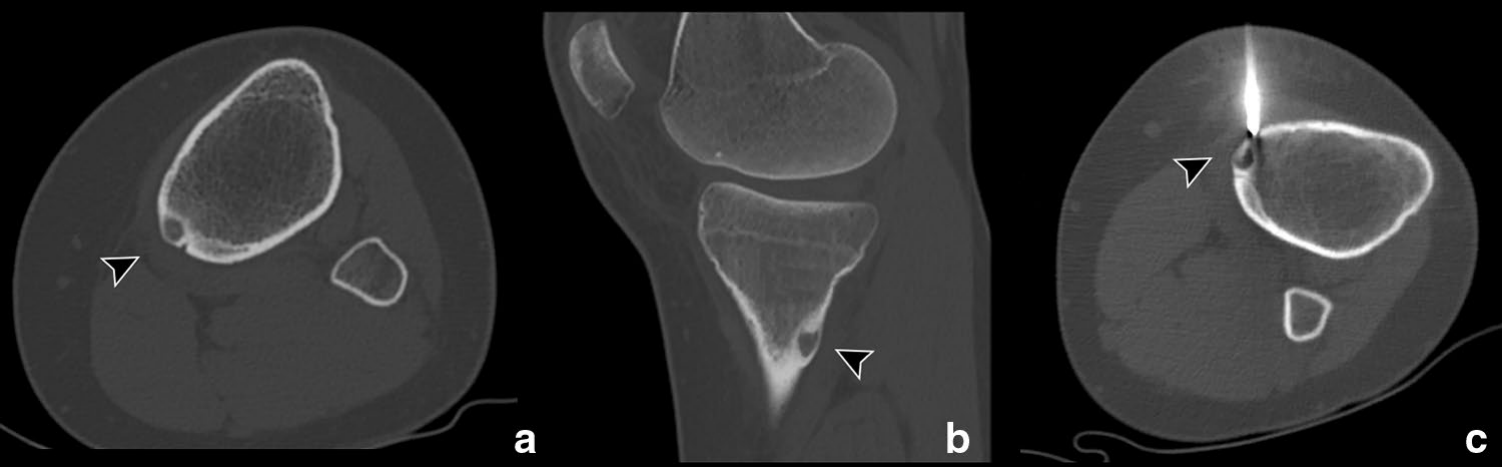
Osteoid osteoma of the left tibial diaphysis. Axial (**a**) and sagittal (**b**) CT scans show the intracortical radiolucent nidus with a central region of mineralization (arrowhead). **c** Axial CT scan performed during RFA procedure shows bone drill/biopsy kit over the cortical surface approaching the nidus

The procedure consists in the insertion of an ablation electrode inside the osteoid osteoma nidus under CT guidance, followed by lesion heating, typically at 90 °C for a time of 5–6 min  (Figs. 1, 2) [15].

The literature also describes ablation techniques involving different combinations of temperature and treatment duration: In particular, some authors have proposed a protocol in which instead of electrode direct overheating at 90 °C, there is an initial phase to raise the temperature to 90 °C, with a 2 min plateau at 60 °C, with a RFA-time longer than 15 min [15–19]. All of the authors achieved a success rate of over 90% and, in particular, Rimondi demonstrated that with this technique success improved from 79 to 98%, recurrences reduced from 21 to 2%, and complications from 5.9 to 0.2% (*p* < 0.001) compared to direct heating at 90 °C with an ablation lasting 4 min [18, 19].

Another RFA technique reported in the literature consisted in an ablative duration of 1–2 min with the electrode temperature at 90 °C, performed on 33 patients monitored for a mean follow-up of 92 months. The procedure was successful for 32 of 33 patients; RFA was repeated in one relapse occurred after 28 months and the patient was then free of symptoms [20].

Other authors described osteoid osteoma ablation performed with 1 cm exposed cool-tip electrode instead of the traditional non-cooled 5 mm electrode on a total of 55 patients, reporting 3 cases of recurrence, 2 of which treated successfully with a second RFA, with disease-free patients for the rest of the follow-up [21, 22].

In a clinical trial on 25 patients, Abboud et al. experimented a dual-cycle RFA (DCRFA) in non-spinal osteoid osteomas, consisting in two consecutive 6 min thermal ablation at 90 °C each separated by electrode cooling at 40 °C. This technique has proved to be 100% successful, recording no recurrences and only one major complication, represented by complex regional pain syndrome of the ankle after DCRFA of a cuboid OO [23].

In a study by Perry et al., OO percutaneous ablation treatment under conventional CT guidance has been compared with cone-beam computed tomography (CBCT) with two-axis fluoroscopic navigational overlay. Twenty-five tumors were treated using CBCT and 35 tumors underwent RFA using conventional CT guidance. The results demonstrated not only that primary clinical success was comparable between the two methods (88.0% for the CBCT group and 88.6% for the conventional CT), but also that the mean effective radiation dose was significantly lower for CBCT compared to CT guidance (0.12 vs. 0.39 mSv, *p* = 0.02) [24].

Akhlaghpoor et al. experimented an osteoid osteoma percutaneous ablation technique based on a combination of radiofrequency and alcohol ablation, consisting in the injection of absolute ethanol into the OO nidus immediately after the extraction of the electrode at the end of the thermal ablation. Fifty-four patients were enrolled, and an immediate pain relief was registered in 52 of 54 patients; recurrence happened in 2 patients and after a second RFA and alcohol ablation complete pain relief was achieved [25].

Recently, Lindquester et al. published a meta-analysis on percutaneous ablation technologies for treatment of osteoid osteoma based on 36 studies, 32 of which (88.9%) evaluated RFA, 3 (8.3%) cryoablation, and 1 (2.8%) microwave (MW) ablation. According to this study, overall success rate of percutaneous techniques was 91.9% (95% confidence interval 91–93%), with success rates of 91.9% for RFA, 91.6% for cryoablation, and 100% for MW, without significant difference when comparing cryoablation and RFA (*p* = 0.92). Among the 36 studies reviewed, 12 studies (33.3%) included patients treated for spinal osteoid osteoma: Success rate could not be determined for all cases, however, for the 9 studies, where patients with spinal OO could be analyzed separately, the overall success rate was 91.6% [26].

Similarly, Lanza et al. [27] performed a systematic review on osteoid osteoma percutaneous thermal ablation techniques evaluating 27 clinical trials where 23 involved RFA, 3 interstitial laser ablation (ILA), and 1 combined RF-ILA. The success rate registered was 90–100%, with a very low complication rate (2%) and a small percentage of patients (5%) who did not respond or experimented recurrence. Results proved that percutaneous thermal ablation remains the treatment of choice compared to surgery; however, authors believed that investigators did not adequately examine treatment failure and recurrence causes in order to obtain statistically significant guidelines for clinical practice; despite this, data analysis suggested that a longer ablation period was related to a lower risk of recurrence [27].

This last evidence is reinforced by a systematic review performed on 69 trials by Tordjman et al. [11] that investigated whether there are factors associated with RFA treatment failures (TF), such as lesion location, ablation time, and patient age and their correlation with the recurrence rate. Results demonstrated that factors like age and osteoid osteoma location were not statistically significantly associated with recurrence rate, while a longer ablation time (> 7 min) was associated with lower TF rate. Another interesting fact emerged from the review is that TF rate evaluated in the studies showed the tendency to decrease over the years between 2011 and 2019 compared to 2002–2010, probably due to progressions in equipment or improvements in operator procedures [11].

A factor that promotes RFA as the treatment of choice for osteoid osteoma is certainly the low recurrence rate, usually attesting in a range from 10 to 16%; nevertheless, in the largest study published conducted by Rimondi on 489 patients treated for non-spinal OO, it dropped to 2% [18].

Data reported in the literature have demonstrated that in case of symptomatic recurrence after the first RFA treatment, re-ablation is almost always resolutive with complete disappearance of symptoms, as suggested by the revision of the literature proposed [10, 16–20, 23–25, ﻿36﻿, ﻿39﻿, ﻿42﻿﻿44﻿, ﻿46﻿, ﻿48﻿﻿50﻿^28^–^43^, ^92^, ^44^–^51^, ^54^–^56^] (Table 2).

**Table 2:** RFA treatment

Lead author	Year	Reference no	RFA minutes	°C	Number of patients	NS/S	Success (%)	Follow-up in months	Number of complications
Abboud	2016	[23]	6	90	25	25/0	100	60	1 M
Akhlaghpoor	2007	[25]	6	90	54	53/1	100	30.5	2 m
Akhlaghpoor	2010	[29]	6	90	21	19/2	100	12	1 m
Albisinni	2017	[16]	15	90	61	0/61	96.7	41.5	2 m
Albisinni	2014	[17]	15	90	27	27/0	100	67.4	0
Baal	2019	[28]	6	90	71	71/0	90.4	29	0
Bourgault	2014	[30]	8	85	87	86/1	97.5	34	1 M, 6 m
Daniilidis	2012	[31]	5	90	29	29/0	89.6	67.2	0
De Palma	2012	[32]	6	90	20	20/0	100	44	0
Doyle	2018	[33]	6	90	32	28/4	100	57	0
Earhart	2013	[34]	6	90	21	21/0	100	17	1 M, 1 m
Esteban Cuesta	2018	[35]	4	90	200	172/28	100	12	0
G Omlor	2012	[36]	7	95	40	39/1	100	35	0
Göksel	2019	[13]	6	90	24	13/0	100	42.5	0
Hage	2018	[37]	5	90	92	91/1	100	95.2	2 M
Hoffmann	2010	[38]	7	90	39	34/5	97.4	32	2 M, 2 m
Lassalle	2017	[39]	5	85	126	126/0	94.3	34,6	5 m
Masciocchi	2015	[92]	7	90	15	15/0	100	24	0
Mastrantuono	2005	[40]	6	90	21	20/1	100	24	0
Morassi	2014	[54]	6	90	13	0/13	100	23,1	0
Neumann	2012	[20]	1–2	95	33	33/0	100	92	0
Neyisci	2019	[41]	6	90	63	63/0	100	19	1 M, 5 m
Niazi	2020	[42]	6	90	34	26/8	100	24	0
Papathanassiou	2011	[43]	6	90	29	27/2	93.1	26.7	1 M, 2 m
Pipola	2020	[56]	15	90	138	0/80	95.5	62.75	0
Rehnitz	2013	[44]	7	90	72	68/4	100	51	0
Rimondi	2012	[18]	15	90	557	557/0	99,6	12	2 M, 3 m
Rimondi	2005	[19]	6	90	97	97/0	97.3	12	1 M, 1 m
Rosenthal	1992	[10]	6	90	263	260/3	91	24	2 m
Sahin	2019	[45]	7	90	116	116/0	100	12	0
Schnapauff	2014	[46]	6–8	90	23	28/5	100	12	0
Seemann	2020	[47]	8	90	33	29/4	100	22.1	0
Tanrıverdi	2020	[48]	5	90	27	27/0	100	46	0
Upadhyay	2017	[49]	3	90	50	48/2	98	12	0
Vanderschueren	2002	[96]	4	90	97	91/6	92	41	1 M, 1 m
Vanderschueren	2009	[55]	4	90	24	0/24	96.5	72	0
Woertler	2001	[50]	5	90	47	46/1	94	22	0
Yuce	2020	[51]	6	90	55	52/3	96.4	22	1 M

*NS* non-spinal osteoid osteoma, *S* spinal osteoid osteoma, *M* major complication requiring treatment, *m* minor complication.

In this regard, in a recent trial, Baal et al. [28] studied 71 individuals trying to identify variables associated with symptomatic recurrence of osteoid osteoma, demonstrating that female gender, younger age below 13 years, and “eccentricity index” (EI) ≥ 3 are possible risk factors for symptomatic recurrence after RF ablation, where EI is calculated by dividing the greatest maximum tumor length by the lowest maximum tumor length from all the anatomic planes.

With reference to spinal osteoid osteomas, they represent 10% of all the cases, mainly affecting the posterior elements [19]. The challenge with the RFA treatment of spinal OO is represented by the risk of injuring neural structures, vessels, and joint facets during the procedure, or by the position of the OO in the posterior part of the vertebral body, so much that for many years they were considered ineligible for RFA [53].

Despite this, the increased use of radiofrequency for spinal osteoid osteoma has given promising results that are substantially comparable to the successes obtained with the non-spinal ones. In particular, many studies have confirmed the safety of CT-guided RFA in the treatment of spinal OO with a success range from 96.5 to 100% [19, 36, 54, 55].

However, in the study by Pipola et al. [56], who compared the results in terms of safety, functional outcome, and recurrence rate of intralesional extracapsular excision (IEE) and RFA in the treatment of spinal OOs, a significantly higher recurrence rate in patients treated with RFA than in ones treated with surgery (12.5 vs. 1.7%) was found. In particular, 58 patients were submitted to an IEE of the tumor and 80 patients to RFA; disease-free survival at the longest follow-up was greater in the surgery group (0.981, 95% confidence interval 0.999–0.963) than in the RFA group (0.841, 95% confidence interval 0.888–0.794) with a statistically significant difference between the two groups (*p* = 0.012).

Although in medical practice, OO ablation procedure success is primarily assessed from a clinical point of view evaluating patient pain regression, MRI role in the follow-up after RFA is well described in the literature [8, 52, 57–61].

From this radiological perspective, the procedure is considered effective whether if immediately after the ablation the nidus is replaced by a necrotic core [52]. This acute post-treatment phase is also associated with inflammatory reaction of the adjacent bone, characterized by hyperemia and edema, this last with a typical ring-like appearance around the ablated zone [57]. Lastly, starting from about a month after the treatment, the site of the nidus is replaced by scarring/fibrotic tissue [52].

Furthermore, MRI investigations during follow-up period document a progressive reduction of the initial perilesional bone marrow edema, whose regression time is very unpredictable, but usually disappearing within 3 months [8, 52, 57–59].

Joint effusion and synovitis are two signs typically associated with intra-articular OO and their rapid regression in few weeks after RFA is easily verifiable through MRI as well [58].

Contrast-enhanced MRI plays a pivotal role, since nidus enhancement not only strongly supports the diagnosis before the procedure, aiding differential diagnosis, but is also a fundamental instrument in the early identification of possible post-treatment disease relapse [58–60].

In this regard, Mahnken et al. [60] proved that contrast enhancement on T1-weighted MRI imaging seems to be predictive of clinically unsuccessful RFA in osteoid osteoma. In their study conducted on 20 patients, a signal-to-noise ratios (SNR) of the nidus before and after contrast administration ≥ 20% after contrast administration appeared to be predictive of symptomatic local tumor recurrence, recommending re-ablation.

Nevertheless, Erbaş et al. [61] conducted a study on 30 patients subjected to osteoid osteoma RFA, evaluating pre- and post- procedural dynamic MRI findings as nidus size, maximum signal intensity (SImax), time of SImax (Tmax), and slope of signal intensity-time (SIT) curves. Results demonstrated that on MR follow-up imaging all patients treated successfully showed a significant decrease in slope of SIT curves and extension of the Tmax values, proving the latter to have the most important positive predictive value for long-term outcomes [61].

Similarly, Rheinheimer et al. [59] assessed the feasibility of diffusion-weighted MRI (DWI) pre- and post-RFA in 10 patients with osteoid osteoma, observing that mean apparent diffusion coefficient (ADC) values significantly increased and contrast enhancement decreased after the treatment, possibly in relation to necrosis and devascularization of the lesion.

MRI is also indicated for the evaluation of post-procedural complications such as osteomyelitis or adjacent soft tissues lesions, especially in those symptomatic patients in which it is necessary to state whether if the pain is due to recurrence/residual disease or to complications developed after treatment [8].

## Interstitial laser ablation (ILA)

Interstitial laser ablation (ILA) is a good choice among percutaneous treatments. An extensive revision of the literature (Table 3) revealed an overall success rate between 94 and 100% for the treatment of osteoid osteoma using ILA [62–70]. Some authors [62, 63] considered this technique easier to use than RFA because it does not require neutral electrodes and there is no current passing through the patient's body. A cost evaluation also showed that ILA is less expensive than RFA [62–65].

**Table 3:** ILA treatment

Lead author	Year	Number of patients	NS/S	Success (%)	Follow-up in month	Number of complications
Gangi	2007	114	102/12	99.1	58.5	1 m
Roqueplan	2009	100	99/1	94	24	1 M, 3 m
Wu	2016	36	36/0	94.4	12	3 m
Etienne	2013	35		94.3	40	4 m
Witt	2000	23	21/2	100	15	3 m
Zouari	2008	15	15/0	100	29	0
Moser	2008	68		98	83	0
Rybak	2010	13	13/0	100	36.5	0
Tsoumakidou	2016	57	57/0	100	12	0

*NS* non-spinal osteoid osteoma, *S* spinal osteoid osteoma, *M* major complication requiring treatment, *m* minor complication.

Gangi et al. [66] reported in a large study ILA performed from June 1994 to June 2004 in 114 patients with a mean age of 22.3 years, with an overall success rate of 99%. During the follow-up period of approximately 58.5 months, six cases of recurrence were described, two of which were related to imprecise needle positioning within the tumor nidus. All these patients were retreated with a second ILA, and only one case was unsuccessful.

Roqueplan [62] evaluated the efficiency and the complication rate of ILA, compared to percutaneous resection. This study reported a 94% of success rate at 24th month in a series of 100 patients, including 1 vertebral OO and 32 intra-articular lesions treated by ILA, and a 95% of success rate in 26 patients treated by percutaneous resection. In the group treated by ILA one procedure was considered technically unsuccessful, while three patients experienced recurrence. Totally four ILA were repeated, two of them failed again. In the second group, only one patient experienced no pain relief.

Similar to Gangi et al. [66], Roqueplan [62] registered that clinical failure was more frequent in patients younger than 16-year old. Low rate of complication was reported in the ILA group, about 4%: one major procedure-related complication due to the accidental injury of peroneal nerve, and three minor ones (hematoma, tendinitis, and infection). In the resection group, it is about 12%, with three minor complication: one case of meralgia paresthetica and cases of skin burns.

In 2016, Wu et al. [67] compared efficiency and complications between ILA and open surgery in a total of 72 patients divided into two groups, containing 36 patients each one. His experience has shown the superiority of percutaneous treatment over surgery, both in terms of clinical success rate (94 vs. 61%) and complication rate (8.3 vs. 27.8%). Despite the limitations of this study due to a limited number of patients and a short follow-up period (12 months), the superiority of percutaneous laser treatment over traditional surgery in terms of efficiency and safety was significant (*p *< 0.05).

Like Roqueplan [62], Etienne et al. [63] reported a clinical success rate about 94% in a series of 35 patients treated by ILA, including one vertebral localization. All procedures were considered technically correct, but two patients did not have pain relief and two patients had a recurrence (rate of 6%). These patients underwent a second procedure and only one had pain relief. Two of the remaining three underwent a third successful procedure. The complication rate in this study was about 5.4% including a skin burn, a patellar enthesopathy, a bone lacuna at the sacro-iliac joint, and a breakage of a needle during percutaneous procedure.

In the study by Witt et al. [68], the success rate was 100% in a group of 23 patients, including one case of recurrence efficiently treated with a second treatment. Only one vertebral lesion was included in the study. During the 15 months follow-up period, three minor complications were reported: Two patients had delayed healing of needle puncture, and one patient with distal phalanx osteoid osteoma lost the nail.

Similarly, Zouari et al. [64] recorded a 100% clinical response in a group of about 15 patients, considering only extremity localizations (hand and foot). In an average of 29 months follow-up, only one patient required second treatment. No minor or major complications occurred; therefore, it seems possible to perform the percutaneous treatment even in the hands and feet with excellent outcomes.

The clinical outcome quoted by Moser et al. [65] also had a high success rate, around 98%, in a cohort of 68 pediatric patients (mean age 12.1 years). During the follow-up period (mean 83 months), five patients had early recurrence in the first six months due to suboptimal ablation of the nidus, two in the next two years. There were no complications related to the procedure, demonstrating that this treatment can be an effective and safe option even in younger patients.

Gangi et al. [66] reported 12 cases of spinal osteoid osteomas effectively ILA-treated without complications. Rybak [69] and Tsoumakidou [70] focused attention exclusively on spinal OOs establishing similar ILA rates of complication (~ 2%) and recurrence (~ 5%) to percutaneous thermal ablation of non-spinal OO. In a series of 17 patients treated with ILA (13 patients) or RFA (4 patients), Rybak reported about 100% of clinical success. Pain relief was achieved in all patients throughout the duration of follow-up (mean 36.5 months) without any major or minor complications. In Tsoumakidou’s review, a total of 61 procedures were conducted in a cohort of 57 patients with vertebral localizations; the second laser ablation was related in three cases to a residual nidus and in one case to a recurrence. A 100% of clinical success was reported without any complication [70].

The literature confirms that ILA is a valid alternative to RFA, although there are still few studies entirely dedicated to spinal lesions [69, 70].

## Microwave ablation (MWA)

Microwave ablation (MWA) is a relatively new technique, which has been growing rapidly in recent years and it provides some unique benefits compared to RFA, specifically less sensitivity to changes in tissue composition and bone impedance reaching higher temperatures within the tumor more rapidly and a more homogeneous volume of thermal ablation zone [71–75].

Moreover, MW provides an important advantage in the manage of those lesions not clearly visible or difficult to reach, since antennas do not need to be necessarily positioned exactly in the center of the nidus for an effective treatment [74].

However, for these same reasons, a recent review performed by Cazzato et al. claimed there are not enough evidences in the literature regarding the safety of MWA treatment on bone tumors, as bone tissue is susceptible to thermo-mediated complications such as secondary fractures [76].

Nowadays there are still not many studies available in the literature about this new technology concerning the treatment of osteoid osteoma (Table 4) [71–75, 77].

**Table 4:** MWA treatment

Lead author	Year	Number of patients	NS/S	Success (%)	Follow-up in month	Number of complications
Kostrzewa	2014	10	10/0	100	6	0
Basile	2014	7	7/0	100	5–13	0
Rinzler	2018	24	24/0	100	1	4 m
Prud'homme	2017	13	13/0	92.3	1	3 m
Reis	2020	15	15/0	92.5	33.8	1 M, 2 m

*NS* non-spinal osteoid osteoma, *S* Spinal osteoid osteoma, *M* major complication requiring treatment, *m* minor complication.

The first two pilot studies published in 2014 are those of Kostrzewa [72] and Basile [73]; both reported 100% technical and clinical success rate over a follow-up period of 5 to 13 months and no complications were observed. However, both studies present the limit of restricted populations, respectively, 10 and 7 patients.

Rinzler et al. [77] published the largest study available in the literature, reporting the results of percutaneous MWA treatment in 24 patients with a 100% of clinical success, and 17% of complication rate, despite the limit of a short follow-up period, only 1-month.

Among the manuscripts available in the literature, only two reported a success rate less than 100%, respectively, the one of Prud'homme et al. with a success rate of 92.3% [74] and Reis et al. with 92% [75]. They studied a population of 13 and 15 patients, respectively, reporting in both cases only one clinical failure. Reis et al. [76] particularly compared the technical success, complication rates, and long-term efficacy or RFA and MWA therapies, considering two groups of 15 patients each. No statistical differences were noted between the group treated by RFA and the one treated by MWA, suggesting a comparable long-term recurrence rate (*p* = 1,0) and complication rates (*p* = 0,60) following RFA and MWA.

Except in Kostrzewa’s work [72], almost all studies reported minor complications, such as local skin numbness and a soft tissue infection in four patients [74], numbness and weakness in the treated area in two patients [75], a self-resolving lesion of a sensory branch of the radial nerve and two skin burn [74]. Reis et al. [75] described a case of a second-degree skin burn at the access site and subsequent cellulitis and repeated infections as a major complication.

In conclusion, there are few reports with an adequate number of patients and follow-ups beyond 6 months, whose results suggest a high clinical success rate and a complete technical success with few complications, albeit more literature evidences are required.

## Cryoablation

Skjeldal et al. [78] were the first to apply cryoablation to an osteoid osteoma in correspondence with the ischial bone, demonstrating an optimal clinical success 1-year after treatment. In 2010, Liu et al. reported two cases of OOs treated by CT-guided cryoablation [79]. Through the years, this procedure has shown to offer advantages in those OOs near nerves, indeed motor nerve regeneration has been demonstrated following cryoablation reversible injury [15, 80]. Another advantage of cryoablation is the ability to identify the ablated zone as a low-density area which corresponds to the ice ball. Real-time monitoring of the ice ball allows to actively control the procedure and to minimize collateral damage to surrounding structures [15, 79, 81, 82].

Recent studies have demonstrated a further beneficial effect of cryoablation promoting an abscopal effect, an immune-specific reaction to tumor cell surrounding the ablation zone [15, 81].

Several trials reported high clinical success rate for percutaneous cryoablation of osteoid osteoma, ranging from 90.5 to 100% [82, 83].

Wu et al. considered six patients with a mean age of 12.6 years, showing a success rate about 100% without any case of recurrence neither complications. Coupal [83] and Santiago [84] considered a larger population of 10 (mean age: 27.9 years) and 21 patients (mean age: 29.9 years), respectively; Coupal et al. [83] reported a 100% of clinical success and no recurrences or complications. Santiago et al. [84] in 21 months of follow-up reported one recurrence and a final clinical success rate about 100% with a second successful cryoablation treatment. Three minor complications (14.3%) were encountered: one mild skin burn and two cases of soft tissue swelling and mechanical pain.

Whitmore [85] reported a 90.5% success rate in a series of 29 child patients (mean age: 11.3 years) reporting two clinical failures. He described two recurrences, both associated with juxtacortical positioning of the cryoprobe, one of them successfully treated by secondary RFA. Minor complication occurred in six patients (21%): three cases of mild dermal blistering, two cases of weakness and pain, and one of transient numbness. All these cases were related to suboptimal positioning of the probe [85].

Only two studies [84, 85] evaluated axial skeleton, even if the study sample was small, one patient and five patients, respectively, with no difference in technical success and response to treatment compared to other lesions.

Percutaneous cryoablation can be considered a clinically effective and safe therapy, considering the low reported complication rate (range from 14.3 to 21%) (Table 5) [82–85] and the ability to provide a direct visualization of the ablation area under CT control (ice ball sign).

**Table 5:** Cryoablation treatment

Lead author	Year	Number of patients	NS/S	Success (%)	Follow-up in month	Number of complications
Wu	2011	6	6/0	100	28.7	0
Coupal	2014	10	10/0	100	24	0
Santiago	2018	21	16/5	100	21	3 m
Whitmore	2016	29	28/1	90.5	12	6 m

*NS* non-spinal osteoid osteoma, *S* Spinal osteoid osteoma, *M* major complication requiring treatment, *m* minor complication.

## Magnetic resonance-guided focused ultrasound (MRg-FUS)

One of the most innovative techniques currently in use is magnetic resonance-guided focused ultrasound (MRg-FUS), which uses high-power ultrasonic energy to thermally coagulate body tissue [86]. It could be considered an attractive alternative to RFA, currently the gold standard.

**Fig. 3:**
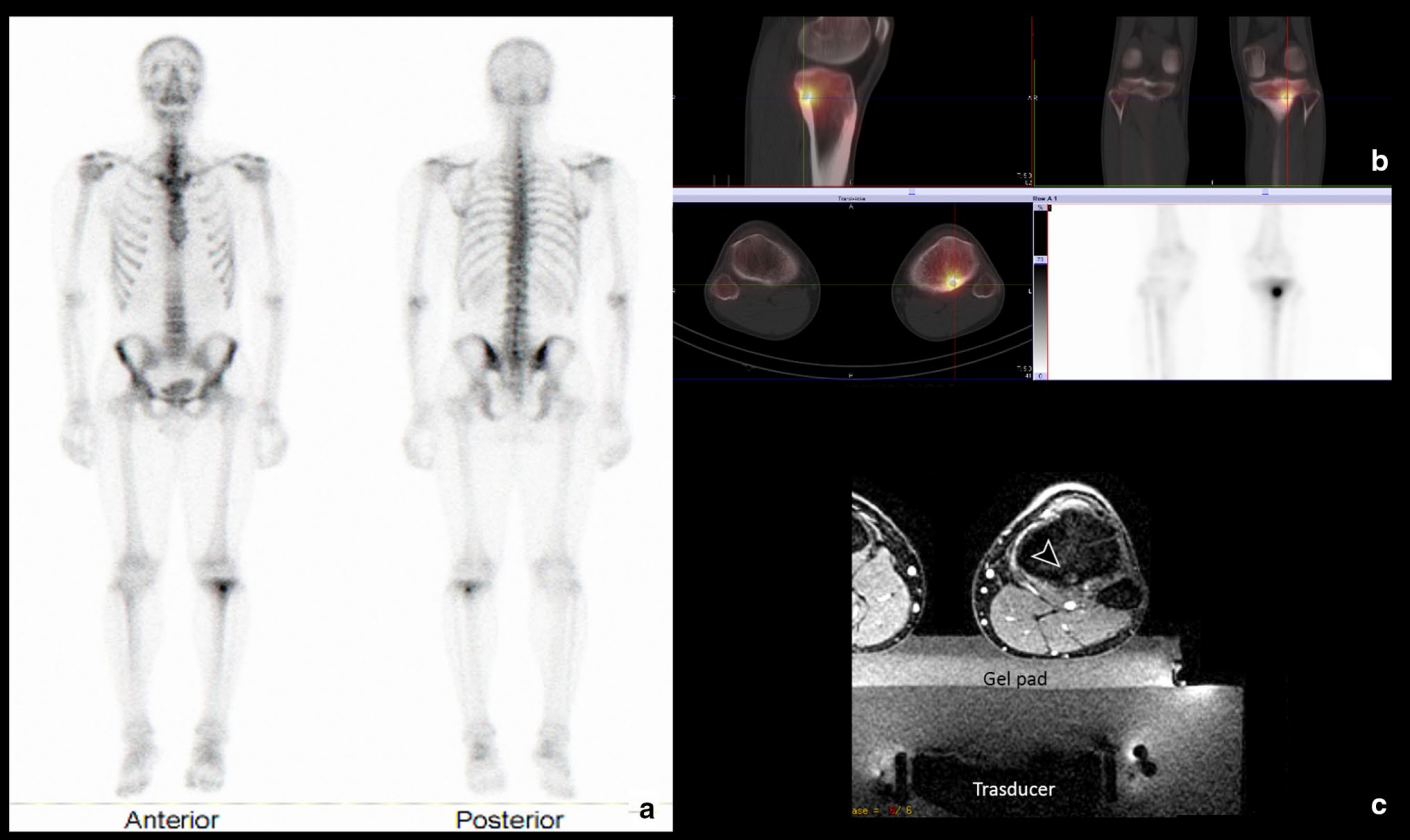
Osteoid osteoma of the left proximal tibial meta-diaphysis. **a** Technetium-99 m bone scintigraphy shows the typical double density sign, characterized by a central focus of intense radiotracer uptake (the nidus) within a region of generalized increase activity. **b** SPECT/CT multiplanar images demonstrate increased radiotracer uptake at the site of the nidus. **c** Axial MRI T2-weighted image with fat saturation during MRg-FUS shows treatment planning and osteoid osteoma location (arrowhead) relative to the transducer

MRg-FUS is a completely non-invasive procedure, based on the conversion of mechanical energy into heat, focused transcutaneously on a small tissue volume, without incisions, thus permitting the periosteal neurolysis and an immediate pain relief [86, 87] (Fig. 3).

Two important advantages are the possibility of high-resolution imaging and the ability to estimate the level of heat generated in the tissue. The latter provides an indirect assessment of the effectiveness of nidus ablation and allows surrounding anatomical structures to be safeguarded by preventing burns [88].

Considering the high prevalence of osteoid osteomas in children and young adults, another relevant aspect concerns non-exposure to radiations and, since there is no mechanical penetration, even the rare risk of fracture or infection [87, 88].

However, accessibility to this new treatment is limited by certain factors, such as lesions not accessible to the MRgFUS system (e.g., spine OO), or general contraindications to perform MRI or administrate contrast medium. Furthermore, this treatment does not allow the biopsy of the lesion. Osteoid osteoma does not always need biopsy if it has a typical appearance. Another technique should be evaluated if an anatomical pathology evaluation is required (not-typical OO appearance) [87].

In literature, the clinical success rate of these new techniques is in a range between 87 and 100% (Table 6). In 2013, Napoli et al. [88] described six patients with osteoid osteoma. During 6 months of follow-up, a 100% of clinical success was reported, without any major or minor complications.

**Table 6:** MRg-FUS treatment

Lead author	Year	Number of patients	NS/S	Success (%)	Follow-up in month	Number of complications
Napoli	2013	6	6/0	100	6	0
Napoli	2017	45	45/0	87	36	0
Geiger	2014	29	29/0	90	12	0
Arrigoni	2019	33	33/0	100	24	0
Masciocchi	2015	15	15/0	93.3	24	0

*NS* non-spinal osteoid osteoma, *S* spinal osteoid osteoma, *M* major complication requiring treatment, *m* minor complication.

In 2017, Napoli et al. [89] described a new trial on a cohort of 45 patients with OO treated with MRg-FUS, in 36 months of follow-up. The overall clinical success rate was about 87% (39 out of 45). In one case, there was no pain relief and a second treatment was required. Of the remaining six patients, three underwent a RFA procedure, while the remaining three, with a partial response, did not underwent a second treatment and were considered clinically failed cases.

Other two studies reported a high rate of clinical response, 90% [84] and 100% [86], respectively. Geiger et al. [90] considered only non-spinal OO in a group of 29 patients (mean age: 25 years) recording three clinical failures in 12 months of follow-up, retracted by surgery or RFA.

Arrigoni et al. [91] described the effectiveness of this treatment in child population, including 33 patients (mean age: 13 years). They reported no final clinical failure during 24 months of follow-up and only one case of recurrence was effectively retreated with a second procedure.

Masciocchi et al. [92] wanted to demonstrate the effectiveness of MRg-FUS comparing with RFA. The study considered 15 patients treated with MRg-FUS and 15 patients treated with RFA. The first group experienced a 93.3% (14 out of 15 patients) clinical response and one clinical failure (6, 6%), while the second one experienced a 100% clinical response. Despite the limited number of patients, no difference was found in the achievement of outcome measures compared to RFA. No complications were found.

According to the literature, MRg-FUS seems to be a valuable alternative option in those cases of typical OO which do not require biopsy. It also represents a safe and well tolerate procedure in those extra-axial lesions that can be reached by MRgFUS system, avoiding radiation exposure.

## Conclusions

In conclusion, RFA is still considered the gold standard procedure for the percutaneous treatment of osteoid osteoma. Although RFA has the possibility to combine treatment with biopsy [15, 83], being a good alternative in cases of non-typical imaging appearance, preliminary biopsy execution is still a matter of debate among the authors, due to negative or inconclusive results. In fact, the usefulness of biopsy in the diagnosis of osteoid osteoma is still unclear, considering that clinical presentation and radiological findings are highly suggestive alone, so that histological examination is seen as unnecessary before therapeutic treatment [89].

Moreover, even if the advent of minimally invasive percutaneous CT-guided procedures made biopsy easier to access prior to intervention, the percentage of diagnostic biopsies reported in the literature remains relatively low (diagnostic yield range of 36–73%) [93–95, 97].

Similar to RFA, ILA and MWA can also be considered as viable percutaneous treatment alternatives with good results. ILA versus RFA is less expensive [52, 57, 58, 62] and presents minor technical concern [52, 57, 58].

MWA has shown promising results and some advantages over RFA such as a more homogeneous thermal ablation volume and a shorter treatment time [72–75]. Moreover, MW antennas do not need to be positioned exactly in the center of the nidus, helping in those lesions not clearly visible or difficult to reach [74].

Among percutaneous treatments, the only one that allows real-time visualization of the ablated zone (ice ball sign) is cryoablation, increasing the safety of treatment [72, 78, 81]. Furthermore, in case of unintended nerve injury, it is associated with lower risk of permanent nerve damage [80].

MRg-FUS is one of the most innovative treatments, the only non-percutaneous one considered in our review. The unquestionable advantage is the lack of radiation, an important factor given the prevalence of OO population [87, 89]. Another benefit is the ability to monitor the temperature in the treated area in real time, avoiding injury to surrounding tissues [88].

However, the main limitation of MRg-FUS is inaccessible locations (such as the spine). Moreover, the significant periosteal reaction that may occur near the osteoid osteoma nidus could be a limit to the US beam penetration, thus making superficial lesion more suitable to treatment [87, 88].

MRg-FUS does not allow the biopsy of the lesion, so in case of non-typical osteoid osteoma appearance, it is not the first-line treatment [87].
